# Association between cardiovascular risk and diastolic blood pressure in older adults with systolic blood pressure less than 130mmHg: a prospective cohort study from 2014 to 2022

**DOI:** 10.1007/s40520-024-02876-7

**Published:** 2024-12-02

**Authors:** Jingjing Hou, Song Zhao, Jie Liu, Xiaoxia Xi, Yawei Xu, Shengfeng Shi, Shikai Yu, Yi Zhang, on behalf of the Northern Shanghai Study investigators

**Affiliations:** 1 Department of Cardiology, Shanghai Tenth People’s Hospital, Tongji University School of Medicine, Yanchang Road 301, Jingan District, Shanghai, 200072 China; 2 Department of Cardiology, Guizhou Provincial People’s Hospital, 83 Zhongshan East Road, Guiyang, 550002 China; 3 Department of Cardiology, Nantong Haimen District People’s Hospital, Beijing West Road 1201, Haimen District, Nantong City, Jiangsu Province China

**Keywords:** Diastolic blood pressure, Isolated diastolic hypertension, Cardiovascular organ damage, Cardiovascular risk, Mortality

## Abstract

**Background:**

The 2017 American College of Cardiology (ACC)/American Heart Association (AHA) guideline lowered the diagnostic threshold for hypertension to a systolic/diastolic blood pressure (SBP/DBP) of 130/80 mmHg. However, the predictive value of DBP for cardiovascular (CV) risk assessment diminishes with aging. The study aimed to explore whether the new diagnostic threshold for diastolic hypertension is associated with increased risk of CV organ damage and major adverse cardiovascular events (MACEs) in older adults.

**Methods:**

1181 individuals aged 65 years or older with SBP < 130 mmHg were enrolled a prospective cohort study. They were classified into Low (< 70 mmHg), Optimal (70 to < 80 mmHg), and High (80 to < 90 mmHg) DBP groups. Cardiac, vascular, and renal organ damage were measured at baseline. The endpoint of the study was MACEs.

**Results:**

Among 1181 participants (average age 71.9 years, 44.8% men), 172 MACEs were observed during an average follow-up of 6.4 years. We found no significant differences in CV organ damage or MACEs rates (Log-rank *P* = 0.73) among three groups. In multivariable Cox regression, compared to the Optimal DBP group, no significant increase in CV risk was observed in the Low DBP group (hazard ratio [HR] 1.02, [95% CI 0.68–1.52], *P* = 0.93) or the High DBP group (HR 1.04, [95% CI 0.72–1.49], *P* = 0.85). Propensity score matching showed consistent results.

**Conclusion:**

In older adults with SBP < 130 mmHg, DBP values 80–89 mmHg were not associated with higher risk of CV organ damage, events or mortality.

**Supplementary Information:**

The online version contains supplementary material available at 10.1007/s40520-024-02876-7.

## Introduction

Since the 2017 American College of Cardiology (ACC)/American Heart Association (AHA) blood pressure guideline lowered the cut-off value for defining stage I hypertension from 140/90 mmHg to 130/80 mmHg [1, 2], numerous studies have been conducted to investigate the rationale and potential effect of this significant change on the management of hypertension [3–10]. A relevant clinical question is whether stage I hypertension (systolic blood pressure [SBP]/diastolic blood pressure [DBP] 130-139/80-89 mmHg) is associated with an increased cardiovascular (CV) risk in real-world older patients. Analyses based on large longitudinal cohorts found that stage I isolated systolic hypertension is significantly associated with increased CV risk across all age groups [3, 9]. However, the association between stage I isolated diastolic hypertension (IDH) and CV risk remains disputed [4–8]. The attenuation of the predictive value of DBP for CV risk with aging may underlie these disputes [11, 12]. Previous studies and meta-analyses demonstrated that the 2017 ACC/AHA-defined IDH was not associated with an increased risk of future CV events in older patients [3, 13], questioning the clinical efficacy and safety of this recommendations in older people. Thus, in the present study, we aimed to investigate whether stage I IDH according to 2017 ACC/AHA recommendations is associated with increased organ damage and CV risk in older people.

## Methods

### Study design and population

The Northern Shanghai Study (NSS) is a prospective cohort study (ClinicalTrial.gov Identifier: NCT02368938) focusing on the CV risk of residents from the communities of the northern area of Shanghai, China. The NSS began in June 2014, detailed descriptions of the study’s fundamental principles and design can be found in our previous publications [14]. The study received approval from the Ethics Committee of Shanghai Tenth People’s Hospital and was financially supported by the Health Youth Talent Project of the Shanghai Municipal Health Commission (2022YQ023) and the Shanghai Municipal Health Commission Clinical Research Project (Youth, 20214Y0152).

After the participants enrollment, clinical examination, anthropometric measurement and a questionnaire will be administered to each participant at baseline and after every 2 years in the follow-up. The last follow-up took place in October 2022, with a median follow-up duration of 6.4 years. All examinations and measurements were conducted in the Shanghai Tenth People’s Hospital.

According to the 2018 European Society of Cardiology hypertension guideline [15] and other research [16], we defined DBP between 70 and 80 mmHg as the Optimal range. Therefore, 1181 participants with SBP < 130 mmHg were classified as follows: Low DBP group (< 70 mmHg), Optimal DBP group (70 to < 80 mmHg), and High DBP group (80 to < 90 mmHg). The groups included both normal and hypertensive patients, with hypertensive patients comprising those undergoing medication treatment. We made the appropriate adjustments in the models. Blood pressure values were measured three times at baseline with a 2-minute interval, using a standardized sitting posture. Organ damage was assessed at baseline by specialized personnel. The primary outcome was major adverse cardiovascular events (MACEs), and results were obtained in October 2022 through data from the insurance system and death registry, combined with telephone follow-up.

### Inclusion and exclusion criteria

The inclusion criteria of our study were (1) age ≥ 65 years, (2) local residents from urban communities in northern Shanghai, and (3) available for long-term follow-up. The exclusion criteria include (1) severe heart failure (New York Heart Association Classification of Heart Failure [NYHA] class IV) or end-stage renal disease, (2) history of stroke within the past 3 months, or (3) malignant tumors with a possible life expectancy of less than 5 years. Informed consent was obtained from each participant.

### Baseline demographic and clinical variables

A standardized questionnaire and face-to-face interviews were used to collect baseline data, including sex, age, education level, lifestyle, smoking and drinking habits, history of hypertension, diabetes, kidney disease, and CV disease, as well as the use of antihypertensive drugs, hypoglycemic drugs, and statin treatment. The trained staff also collected anthropometric measurements, including height, weight, hip circumference, and waist circumference from the participants.

Blood pressure was measured in a quiet room after 5 minutes of rest, using a sphygmomanometer (Omron Healthcare, Kyoto, Japan). Three consecutive measurements were obtained at 2-minute intervals with participants in a sitting position. The average of the second and third blood pressure measurements was considered for analysis.

The laboratory medicine department of Shanghai Tenth People’s Hospital conducted a thorough analysis of the blood and urine samples from each participant. The modified modification of diet in renal disease (MDRD) formula [17] was used to calculate the estimated glomerular filtration rate (e-GFR). All measurements were carried out by well-trained staff.

### Outcomes of interest

All organ damage statistics were assessed at baseline. The left ventricular mass index (LVMI), the ratio of peek early diastolic transmitral flow velocity (E) and the early diastolic lateral mitral annular velocity (Ea) [E/Ea], and carotid artery intima-media thickness (CIMT) were evaluated using a MyLab 30 CV machine (ESAOTE SPA, Italy). Carotid-femoral pulse wave velocity (CF-PWV) was measured with the SphygomoCor equipment (AtCor, Australia), while the ankle brachial index (ABI) was assessed using the VP-1000 equipment (Omron, Japan). Renal dysfunction was evaluated using the urinary albumin-creatinine ratio (UACR) and estimated glomerular filtration rate (e-GFR), and they can be calculated individually using the following formulas: UACR = [urinary microalbumin (mg/dl)/urinary creatinine (μmol/l)] × 8840, e-GFR (ml/ (min × 1.73 m^2^)) =175 × (Scr)^-1.154^ × (age)^-0.203^ × [0.742 (if female)]

${\text{UACR }} = \, [{\text{urinary microalbumin}}\left( {{\text{mg}}/{\text{dl}}} \right) \, /{\text{ urinary creatinine}}\left( {\mu {\text{mol}}/{\text{l}}} \right)] \, \times { 884}0,{\text{e - GFR }}({\text{ml}}/({\text{min}} \times {1}.{\text{73m}}^{{2}} )) \, = {175} \times \left( {{\text{Scr}}} \right)^{{ - {1}.{154}}} \times \left( {{\text{age}}} \right)^{{ - 0.{2}0{3}}} \times \, \left[ {0.{742 }\left( {\text{if female}} \right)} \right].$ In this formula, Scr refers to serum creatinine levels (mg/dl) [17, 18].

In summary, CV organ damage includes cardiac, vascular, and renal damage. Left ventricular hypertrophy (LVH) was defined as LVMI ≥ 115 g/m^2^ in males or LVMI ≥ 95 g/m^2^ in females [19]. Left ventricular diastolic dysfunction (LVDD) was characterized by E/Ea ≥ 15, or 15 > E/Ea > 8, in conjunction with any of the following: LVMI > 149 g/m^2^ in males or LVMI ≥ 122 g/m^2^ in females [20]. Vascular damage included the presence of carotid hypertrophy, arterial stiffness (AS), and peripheral arterial disease (PAD). Carotid hypertrophy was identified by CIMT > 0.9mm [21], AS was indicated by CF-PWV ≥ 12 m/s, and PAD was classified as ABI < 0.9 [22]. Renal damage was defined as e-GFR < 60 ml/min/1.73 m^2^ or UACR > 30mg/mmol [23].

### Clinical outcomes

The primary outcome was defined as a composite endpoint of MACEs, including nonfatal acute myocardial infarction, non-fatal stroke, and death. The vital status and CV events of all participants were confirmed by reviewing the Shanghai medical insurance system and the Shanghai morality record system, combined with telephone follow-up in October 2022. Outcomes were determined by an independent, blinded adjudication committee, which included experts from cardiology and neurology. If a participant had more than one event during follow-up, the first event was counted as the outcome.

### Statistical analysis

Continuous variables were presented as mean ± standard deviation for normally distributed data or median (interquartile range) for skewed data. Categorical variables were presented as absolute numbers and percentages in parentheses. Participants were divided into three groups according to the levels of DBP (< 70 mmHg, 70 to < 80mmHg, 80 to < 90mmHg). Due to the limited sample size of individuals with DBP ≥ 90 mmHg (n = 32, number of events = 3), many analyses were not performed in this group. Differences in continuous and categorical variables among the groups were assessed using one-way ANOVA (or Kruskal-Wallis test for non-normal distributed variables) and chi-square tests, respectively. Logistic regression models were performed to evaluate the association between CV organ damage and DBP groups. Three models were constructed: (1) an unadjusted model; (2) a model adjusted for age, body mass index (BMI), sex, history of CV disease, diabetes, SBP, current smoking, and the triglyceride/high-density lipoprotein ratio; and (3) an extended model that additionally adjusted for antihypertensive, statin, and hypoglycemic treatments, in addition to the covariates included in model (2). Survival analysis was performed to explore the survival rates of MACEs across the DBP groups. Multivariable Cox proportional hazards regression was conducted to determine the association between MACEs and DBP groups, adjusting for age, BMI, sex, history of CV disease, diabetes, SBP, current smoking, triglycerides/high-density lipoprotein ratio, e-GFR, and treatments with antihypertensive, statin, and hypoglycemic. In order to minimize potential bias, we conducted a 1:1 propensity score matching (PSM) analysis between the Optimal DBP group and the High DBP group. A two-tailed P-value < 0.05 was considered significant. All statistical analyses were performed using R version 4.2.3.

## Results

### Characteristics of Participants

The characteristics of the participants and their comparisons among the three groups are presented in Table 1. The average age of the participants was 71.9 years, with 53.4% classified as hypertensive, 18.9% having diabetes, 34.2% with coronary heart disease, and 19.8% having a history of stroke. The High DBP group showed the highest SBP and DBP, the highest prevalence of PAD, but the lowest pulse pressure and the lowest prevalence of microalbuminuria (MAU). During the median follow-up of 6.4 years, the primary outcome occurred in 172 participants (14.6%), including 20 (1.7%) nonfatal acute myocardial infarction, 52 (4.4%) nonfatal stroke, and 108 (9.1%) all cause death. No significant differences in the rates of CV events were observed among the three DBP groups.

**Table 1:** Baseline characteristics of study participants

Variables	Overall (n = 1181)	Low DBP (< 70 mmHg) (n = 251)	Optimal DBP (70 to < 80 mmHg) (n = 565)	High DBP (80 to < 90 mmHg) (n = 365)	*P*
Age, years	71.9 ± 6.1	71.8 ± 5.8	71.9 ± 6.0	72.0 ± 6.6	0.83
Men, n (%)	529(44.8%)	119(47.4%)	247(43.7%)	163(44.7%)	0.62
Smokers, n (%)	273(23.1%)	60(23.9%)	136(24.1%)	77(21.2%)	0.55
Body mass index, kg/m^2^	24.3 ± 3.4	23.9 ± 3.4	24.4 ± 3.4	24.6 ± 3.5	0.048*
SBP, mmHg	118.1 ± 7.7	113.8 ± 8.8	118.1 ± 7.4	121.2 ± 5.6	< 0.01*
DBP, mmHg	74.1 ± 7.3	63.7 ± 4.6	73.7 ± 3.3	82.1 ± 2.5	< 0.01*
Pulse pressure, mmHg	44.0 ± 8.5	50.1 ± 9.8	44.4 ± 7.5	39.2 ± 5.5	< 0.01*
Serum glucose, mmol/l	5.69 ± 1.75	5.83 ± 1.71	5.69 ± 1.73	5.61 ± 1.81	0.30
Serum creatinine, μmol/l	74.83 ± 19.38	75.09 ± 18.23	74.54 ± 19.72	75.11 ± 19.66	0.88
HDL, mmol/l	1.41 ± 0.36	1.43 ± 0.36	1.41 ± 0.37	1.40 ± 0.36	0.65
LDL, mmol/l	3.22 ± 0.86	3.18 ± 0.96	3.28 ± 0.82	3.16 ± 0.85	0.09
Triglycerides, mmol/l	1.55 ± 0.84	1.50 ± 0.77	1.59 ± 0.91	1.52 ± 0.75	0.35
Total cholesterol, mmol/l	5.23 ± 1.01	5.16 ± 1.09	5.31 ± 0.98	5.16 ± 1.00	0.04*
TC/HDL ratio	3.90 ± 1.07	3.79 ± 1.11	3.97 ± 1.08	3.86 ± 1.02	0.06
Hypertension, n (%)	631(53.4%)	142(56.6%)	296(52.4%)	193(52.9%)	0.53
Diabetes, n (%)	223(18.9%)	64(25.5%)	95(16.8%)	64(17.5%)	0.01*
Stroke, n (%)	223(19.8%)	44(17.7%)	110(19.5%)	79(21.6%)	0.47
Coronary heart disease, n (%)	404(34.2%)	83(33.1%)	201(35.6%)	120(32.9%)	0.64
Antihypertensive drugs, n (%)	599(50.7%)	136(54.2%)	280(49.6%)	183(50.1%)	0.46
Statin treatment, n (%)	98(8.3%)	27(10.8%)	49(8.7%)	22(6.0%)	0.10
Hypoglycemic drugs, n (%)	184(15.6%)	53(21.1%)	82(14.5%)	49(13.4%)	0.02*
Renal disease, n (%)	94(8.0%)	39(6.9%)	21(8.4%)	34(9.3%)	0.40
Organ damage					
LVMI, g/m^2^	90.01 ± 29.39	89.68 ± 29.63	90.10 ± 28.11	90.09 ± 31.20	0.98
CIMT, um	600(510–710)	620(520–720)	600(510–700)	600(513–710)	0.42
Ankle-brachial index	1.07(0.98–1.14)	1.09(1.01–1.15)	1.07(0.98–1.14)	1.05(0.95–1.12)	< 0.01*
e-GFR, ml/min/1.73m^2^	78.08(68.45–88.96)	78.47(66.61–89.09)	78.47(68.88–88.97)	76.96(68.25–88.01)	0.78
CF-PWV, m/s	9.10(8.00–10.60)	9.10(8.00–10.80)	9.20(8.00–10.70)	9.00(7.80–10.30)	0.28
E/Ea	10.14 ± 3.75	10.67 ± 3.88	10.15 ± 3.61	9.75 ± 3.84	0.01*
UACR	24.21(14.01–47.10)	26.96(16.21–51.31)	23.97(14.13–49.75)	21.76(12.41–39.00)	< 0.01*
LVH, n (%)	319(27.2%)	61(24.5%)	153(27.3%)	105(28.9%)	0.48
LVDD, n (%)	109(9.5%)	32(13.2%)	50(9.1%)	27(7.6%)	0.07
Carotid hypertrophy, n (%)	62(5.3%)	14(5.6%)	28(5.0%)	20(5.5%)	0.92
Arterial stiffness, n (%)	139(12.2%)	30(12.3%)	67(12.2%)	42(12.1%)	0.99
PAD, n (%)	126(10.9%)	18(7.3%)	57(10.3%)	51(14.4%)	0.02*
MAU, n (%)	448(39.4%)	109(45.0%)	225(41.3%)	114(32.7%)	< 0.01*
Renal damage, n (%)	141(12.1%)	27(10.9%)	71(12.7%)	43(11.8%)	0.77
Outcomes					
MACEs, n (%)	172(14.6%)	41(16.5%)	80(14.2%)	51(14.0%)	0.62
Nonfatal MI, n (%)	20(1.7%)	6(2.4%)	9(1.6%)	5(1.4%)	0.60
Nonfatal stroke, n (%)	52(4.4%)	15(6.0%)	26(4.6%)	11(3.0%)	0.19
Cardiovascular death, n (%)	44(3.7%)	6(2.4%)	23(4.1%)	15(4.1%)	0.45
All cause death, n (%)	108(9.1%)	23(9.2%)	51(9.0%)	34(9.3%)	0.99

Values are represented as n (%) or mean ± SD / median (interquartile range). Low DBP group (< 70 mmHg), Optimal DBP group (70 to < 80 mmHg), and High DBP group (80 to < 90 mmHg)

*SBP/DBP* systolic/diastolic blood pressure, *HDL* high-density lipoprotein, *LDL* low-density lipoprotein, *TC/HDL* total cholesterol/ high-density lipoprotein, *LVMI* left ventricular mass index, *CIMT* carotid intima-media thickness, *e-GFR* estimated glomerular filtration rate, *CF-PWV* carotid-femoral pulse wave velocity, *E/Ea* ratio of peek early diastolic transmitral flow velocity (E) and the early diastolic lateral mitral annular velocity (Ea), *UACR* urinary albumin-creatinine ratio, *LVH* left ventricular hypertrophy, *LVDD* left ventricular diastolic dysfunction, *PAD* peripheral arterial disease, *MAU* microalbuminuria, *MACEs* major adverse cardiovascular events, *MI* myocardial Infarction

^*^P < 0.05

### DBP and CV organ damage

As shown in Table 2, We conducted a cross-sectional study at baseline using logistic regression to explore the relationship between CV organ damage and DBP. Using the Optimal DBP group as a reference, the results of the regression analysis (Model 1) indicated that neither the Low DBP group nor the High DBP group had significant associations with cardiac, vascular, or renal organ damage, except for the High DBP group, which showed a decreased odds ratio (OR) for MAU (OR 0.69, [95%CI 0.52–0.91], *P* = 0.01). After adjusting for age, BMI, sex, history of CV disease, diabetes, SBP, current smoking, and triglycerides/high-density lipoprotein ratio (Model 2), neither the Low DBP group nor the High DBP group demonstrated any significant associations with cardiac, vascular, or renal organ damage. In Model 3, which incorporated adjustments for antihypertensive treatment, statin treatment, and hypoglycemic treatment in addition to the variables in Model 2, similar results were observed.

**Table 2:** Association of cardiovascular organ damage with DBP analyzed by logistic regression

		Model 1			Model 2			Model 3		
		Optimal DBP	Low DBP	High DBP	Optimal DBP	Low DBP	High DBP	Optimal DBP	Low DBP	High DBP
Cardiac organ damage										
LVH	OR(95%CI)	ref	0.86(0.61–1.22)	1.08(0.81–1.45)	ref	0.88(0.60–1.27)	1.18(0.86–1.61)	ref	0.87(0.60–1.26)	1.16(0.85–1.60)
	*P*	–	0.40	0.60	–	0.48	0.32	–	0.47	0.35
LVDD	OR(95%CI)	–	1.51(0.94–2.42)	0.82(0.51–1.34)	–	1.25(0.75–2.08)	0.93(0.56–1.54)	–	1.24(0.74–2.05)	0.92(0.55–1.53)
	*P*	–	0.09	0.44	–	0.39	0.77	–	0.42	0.75
Vascular organ damage										
Carotid hypertrop-hy	OR(95%CI)	–	1.13(0.58–2.18)	1.11(0.61–1.99)	-	0.93(0.45–1.94)	1.31(0.71–2.42)	–	0.93(0.44–1.95)	1.33(0.72–2.48)
	*P*	–	0.72	0.74	–	0.84	0.39	–	0.85	0.36
AS	OR(95%CI)	–	1.02(0.64–1.61)	0.99(0.66–1.50)	–	0.95(0.58–1.56)	1.05(0.68–1.64)	–	0.95(0.58–1.56)	1.05(0.67–1.63)
	*P*	–	0.95	0.97	-	0.83	0.82	–	0.83	0.84
PAD	OR(95%CI)	–	0.68(0.39–1.19)	1.46(0.97–2.18)	-	0.74(0.40–1.38)	1.38(0.89–2.14)	–	0.74(0.40–1.38)	1.38(0.89–2.13)
	*P*	–	0.18	0.07	-	0.34	0.15	–	0.35	0.15
Renal organ damage										
Renal damage	OR(95%CI)	–	0.82(0.52–1.34)	0.92(0.61–1.37)	-	0.85(0.50–1.42)	0.97(0.62–1.51)	–	0.85(0.50–1.42)	0.99(0.63–1.53)
	*P*	–	0.48	0.69	–	0.55	0.91	–	0.54	0.96
MAU	OR(95%CI)	–	1.17(0.86–1.58)	0.69(0.52–0.91)	–	0.98(0.70–1.36)	0.79(0.59–1.07)	–	0.97(0.70–1.36)	0.79(0.59–1.07)
	*P*	–	0.33	0.01*	–	0.89	0.13	–	0.87	0.13

Model 1 without adjustment; Model 2 with adjustment for age, body mass index, sex, cardiovascular disease history, diabetes, systolic blood pressure, current smoking, triglycerides/high-density lipoprotein ratio; Model 3 including additional adjustments for antihypertensive treatment, statin treatment, and hypoglycemic treatment in addition to the variables in Model 2. Low DBP group (< 70 mmHg), Optimal DBP group (70 to < 80 mmHg), and High DBP group (80 to < 90 mmHg)

*DBP* diastolic blood pressure, *LVH* left ventricular hypertrophy, *LVDD* left ventricular diastolic dysfunction, *AS* arterial stiffness, *PAD* peripheral arterial disease, *MAU* microalbuminuria, *OR* odds ratio, *CI* confidence interval

^*^P < 0.05

### DBP and MACEs

**Fig. 1:**
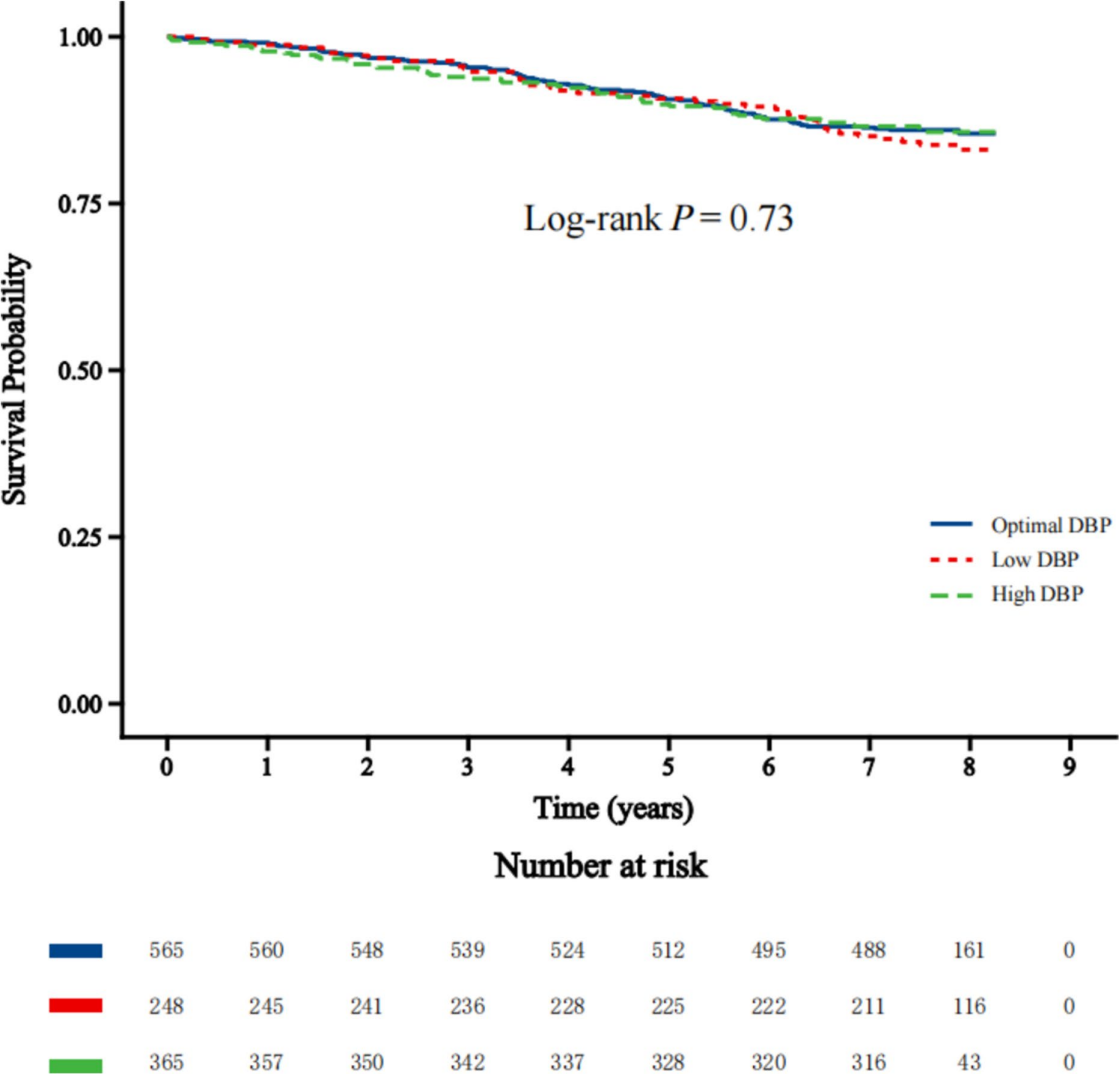
Survival rates of MACEs with DBP. *MACEs* major adverse cardiovascular events, *DBP* diastolic blood pressure. MACEs consisted of all cause death, nonfatal myocardial infarction, and nonfatal stroke. Low DBP (< 70 mmHg), Optimal DBP (70 to < 80 mmHg), High DBP (80 to < 90 mmHg)

**Fig. 2:**
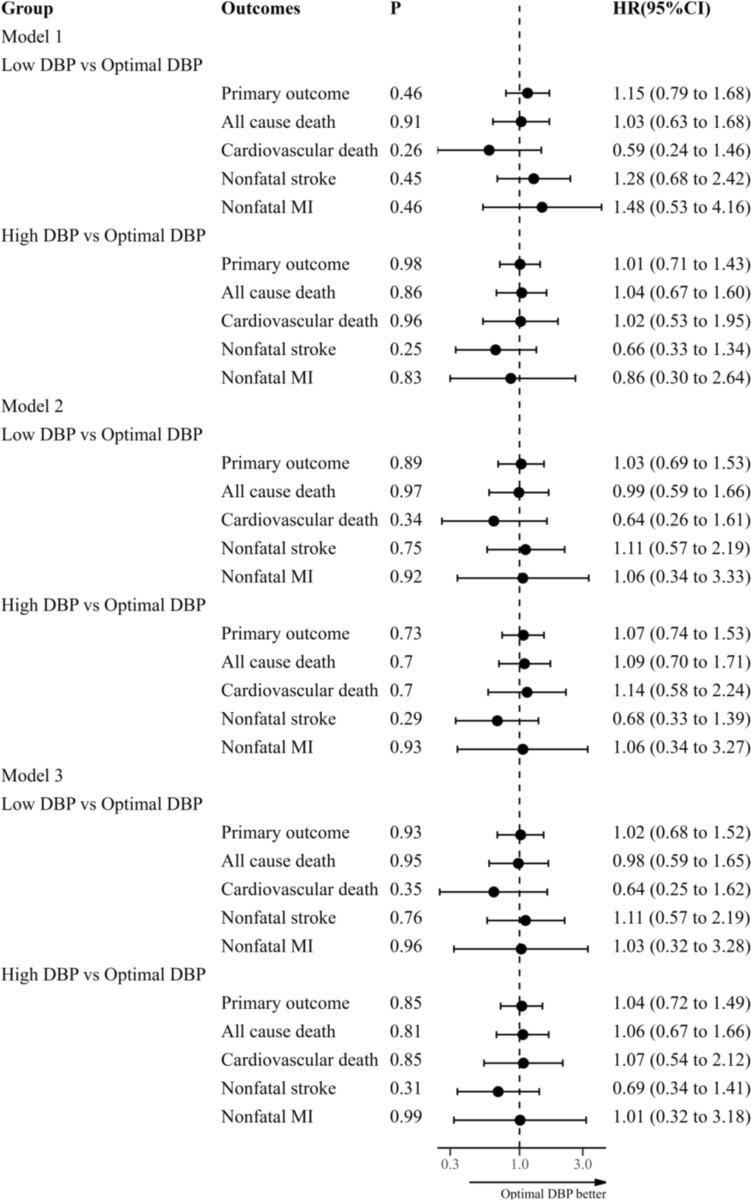
Association of MACEs with DBP analyzed by Cox proportional hazards regression. *MACEs* major adverse cardiovascular events, *DBP* diastolic blood pressure, *MI* myocardial infarction, *HR* hazard ratio, *CI* confidence interval. MACEs consisted of all cause death, nonfatal myocardial infarction, and nonfatal stroke; Model 1 without adjustment; Model 2 with adjustment for age, body mass index, sex, cardiovascular disease history, diabetes, systolic blood pressure, current smoking, triglycerides/high-density lipoprotein ratio. Model 3 including additional adjustments for estimated glomerular filtration rate, antihypertensive treatment, statin treatment, and hypoglycemic treatment in addition to the variables in Model 2. Low DBP (< 70 mmHg), Optimal DBP (70 to < 80 mmHg), High DBP (80 to < 90 mmHg)

As illustrated in the Kaplan-Meier curve (Fig. 1), survival analysis indicated no significant difference in the survival rates of MACEs among the three groups (Log-rank* P* = 0.73). Furthermore, as shown in Fig. 2, when using the Optimal DBP group as a reference, the Cox proportional hazards regression analysis (Model 1) showed no significant associations between the MACEs and either the Low DBP group or the High DBP group. Similar results were observed after adjustments for the covariates mentioned above (Model 2 and Model 3).

To mitigate potential bias and balance the covariates, a 1:1 propensity score matching (PSM) analysis was conducted between the Optimal DBP group and the High DBP group. A total of 730 patients were included, with each subgroup comprising 365 patients. Survival analysis and multivariate Cox proportional hazards regression revealed no significant associations between MACEs and the PSM groups, consistent with previous findings (Table S1, Fig. S1, and Fig. S2).

## Discussion

In this study on older Chinese people with SBP < 130 mmHg, we demonstrated that compared with DBP < 80mmHg, DBP between 80 and 90 mmHg was not significantly associated with higher risk of CV organ damage or events, thereby questioning the clinical validity of this lower DBP target in older patients.

The association between 2017 ACC/AHA-defined IDH and the incidence of CV events have drawn considerable attention following the release of the 2017 ACC/AHA hypertension guideline. McEvoy JM et al. [6] found no correlation between IDH and CV events in the Atherosclerosis Risk in Communities (ARIC) study among participants with a mean age of 56 years. McGrath BP et al. [24] revealed that in a population over the age of 55, there was no significant association between IDH and CV events in a United Kingdom (UK) biobank sample of 151,831 participants over a 10-year period. Additionally, Wu S et al.[7] reported no correlation between IDH and CV events among individuals aged 48 and over. In contrast to previous studies that involved older participants, the results from 1,746,493 individuals in the Japan Medical Data Center (JMDC), with a mean age of 42.9 years, showed that IDH was associated with an increased risk of CV disease [5]. Furthermore, other studies investigating the relationship between 2017 ACC/AHA-defined IDH and CV risk in young people have also yielded similar results. Lee H et al. [8] revealed that individuals aged 20-39 in South Korea experienced a significant association between IDH and CV events after 13.2 years of follow-up. The Chicago Heart Association Detection Project Industry study, which included 39,441 participants aged 18-49, also found a significant association between IDH and CV events [25]. Overall, there seems to be a trend in current large cohort studies indicating that the 2017 ACC/AHA-defined IDH is associated with CV events in the young population, whereas this relationship does not seem to exist in the elderly population.

Similarly, our study, which specifically focusing on elderly Chinese individuals, again demonstrated the lack of association between 2017 ACC/AHA-defined IDH and CV events. The observed lack of significant association between IDH and CV risk in the elderly population may be attributed to the impact of increased vascular stiffness and reduced recoil force [26], which tend to result in lower DBP values among older adults [27]. Consequently, there are too few individuals with sufficiently high DBP to contribute to an increased incidence of CV events. In our study, individuals with DBP greater than 90 mmHg comprised only 32 participants, with a mere 3 observed events. Another possible explanation for this null association could be the regression to the mean phenomenon. If DBP measurements were repeated in individuals with IDH, the values would likely approach the mean DBP, leading in reclassification as having normal blood pressure [28].

Another group that warrants attention is individuals with DBP less than 60 mmHg. McEvoy JW et al. reported that people with low DBP (less than 60 mmHg) are more likely to suffer from coronary heart disease and mortality [29]. However, numerous recent clinical studies have revealed that lower blood pressure is associated with enhanced CV benefits, and evidence against the J-curve phenomenon is becoming increasingly substantial. Mendelian Randomization analyses have revealed a linear correlation between DBP and CV events, with no evidence of J- or U-shaped relationships [30]. Furthermore, low DBP level do not appear to contribute to an increase in CV events [31], which aligns with our study findings, as we observed no elevated CV risk in participants with lower DBP. Overall, these findings suggest that DBP has limited clinical value in predicting future CV risk in older adults.

The results of this study should be interpreted under its limitations. Firstly, we could not extensively analyze the CV risk of participants with DBP greater than 90 mmHg due to the very small sample size (n = 32). Secondly, our investigation into the association between DBP and CV organ damage was based on cross-sectional data, which limits our ability to infer causality. Thirdly, participants’ medical conditions and medication use were self-reported through questionnaires, which may potentially introduce biased information. Although we adjusted for medication use in the model, this adjustment may not fully account for the effects of the medications.

## Conclusions

In summary, no increased CV organ damage or risk was observed in older adults with stage I IDH defined by the new cut-off value of 80 mmHg.

## Supplementary Information

Below is the link to the electronic supplementary material.

Supplementary file1 (DOCX 420 KB)

## Data Availability

The data that support the findings of this study are available on request from the corresponding author, upon reasonable request.
