# Supplementary material for: Association between cardiovascular risk and diastolic blood pressure in older adults with systolic blood pressure less than 130mmHg: a prospective cohort study from 2014 to 2022
Source: Aging Clin Exp Res. 2024 Dec 2;36(1):229. doi: 10.1007/s40520-024-02876-7 (PMC11611959; doi:10.1007/s40520-024-02876-7)
Supplement: Supplementary file 1 — Supplementary file1 (DOCX 420 KB) [file 40520_2024_2876_MOESM1_ESM.docx]

**Table S1** Baseline characteristics of participants after propensity score matching

| Characteristic | Before matching | | SMD^△^ | After matching | | SMD^△^ |
| --- | --- | --- | --- | --- | --- | --- |
|  | Optimal DBP, N = 565 | High DBP, N = 365 |  | Optimal DBP, N = 365 | High DBP, N = 365 |  |
| Age, years | 71.9(6.0) | 72.0 (6.6) | -0.014 | 72.0 (5.9) | 72.0 (6.6) | -0.001 |
| Men, n (%) | 247 (43.7%) | 163 (44.7%) | 0.019 | 157 (43.0%) | 163 (44.7%) | 0.033 |
| Smokers, n (%) | 136 (24.1%) | 77 (21.2%) | -0.073 | 92 (25.2%) | 77 (21.2%) | -0.101 |
| Body mass index, kg/m^2^ | 24.4 (3.4) | 24.6 (3.5) | 0.054 | 24.4 (3.4) | 24.6 (3.5) | 0.057 |
| SBP, mmHg | 118.1 (7.4) | 121.2 (5.6) | 0.556 | 119.3(6.9) | 121.2 (5.6) | 0.347 |
| DBP, mmHg | 73.7 (3.3) | 82.1 (2.5) | 3.306 | 75.6 (2.6) | 82.1 (2.5) | 2.542 |
| Pulse pressure, mmHg | 44.4 (7.5) | 39.2 (5.5) | -0.954 | 43.7 (7.2) | 39.2 (5.5) | -0.816 |
| Serum glucose, mmol/l | 5.7 (1.7) | 5.6 (1.8) | -0.042 | 5.7 (1.7) | 5.6 (1.8) | -0.043 |
| Serum creatinine, umol/l | 74.5 (19.7) | 75.1 (19.7) | 0.034 | 75.1 (20.4) | 75.1 (19.7) | 0.013 |
| HDL, mmol/l | 1.4 (0.4) | 1.4 (0.4) | -0.007 | 1.42 (0.37) | 1.4 (0.4) | -0.038 |
| LDL, mmol/l | 3.3 (0.8) | 3.2 (0.9) | -0.141 | 3.3 (0.8) | 3.2 (0.9) | -0.095 |
| Triglycerides, mmol/l | 1.6 (0.9) | 1.5 (0.8) | -0.086 | 1.6 (0.9) | 1.5 (0.8) | -0.051 |
| Total cholesterol, mmol/l | 5.3 (1.0) | 5.2 (1.0) | -0.150 | 5.3 (1.0) | 5.2 (1.0) | -0.104 |
| TC/HDL ratio | 4.0 (1.1) | 3.9 (1.0) | -0.119 | 3.9 (1.1) | 3.9 (1.0) | -0.059 |
| LVMI, g/m^2^ | 90.0 (28.0) | 90.5 (32.1) | 0.016 | 89.2 (26.5) | 90.5 (32.1) | 0.042 |
| CIMT, um | 625.7(155.3) | 629.6(155.4) | 0.025 | 622.9. (151.4) | 629.6(155.4) | 0.043 |
| Ankle-brachial index | 1.06(0.13) | 1.04 (0.12) | -0.185 | 1.06. (0.13) | 1.04 (0.12) | -0.162 |
| e-GFR, ml/min/1.73m^2^ | 78.8(16.8) | 78.8(24.8) | 0.002 | 78.2 (16.7) | 78.8 (24.8) | 0.024 |
| CF-PWV, m/s | 9.5(2.2) | 9.3(2.2) | -0.085 | 9.4 (2.3) | 9.3 (2.2) | -0.046 |
| E/Ea | 10.1 (3.6) | 9.8 (3.8) | -0.105 | 10.0(3.7) | 9.8(3.8) | -0.057 |
| UACR | 54.0(97.0) | 48.1(103.6) | -0.058 | 53.4(96.8) | 48.1 (103.6) | -0.052 |
| LVH, n (%) | 153 (27.3%) | 105 (28.9%) | 0.037 | 97 (26.6%) | 105 (28.9%) | 0.048 |
| LVDD, n (%) | 50 (9.1%) | 27 (7.6%) | -0.055 | 33 (9.0%) | 27 (7.6%) | -0.063 |
| Carotid hypertrophy, n (%) | 28 (5.0%) | 20 (5.5%) | 0.023 | 17 (4.7%) | 20 (5.5%) | 0.036 |
| Arterial stiffness, n (%) | 67 (12.2%) | 42 (12.1%) | -0.011 | 41 (11.2%) | 42 (12.1%) | 0.009 |
| PAD, n (%) | 57 (10.3%) | 51 (14.4%) | 0.112 | 39 (10.7%) | 51 (14.4%) | 0.095 |
| MAU, n (%) | 225 (41.3%) | 114 (32.7%) | -0.185 | 155 (42.5%) | 114 (32.7%) | -0.242 |
| Renal damage, n (%) | 71 (12.6%) | 43 (11.8%) | -0.024 | 45 (12.3%) | 43 (11.8%) | -0.017 |
| Hypertension, n (%) | 296 (52.4%) | 193 (52.9%) | 0.010 | 191 (52.3%) | 193 (52.9%) | 0.011 |
| Diabetes, n (%) | 95 (16.8%) | 64 (17.5%) | 0.019 | 63 (17.3%) | 64 (17.5%) | 0.007 |
| Stroke, n (%) | 110 (19.5%) | 79 (21.6%) | 0.053 | 73 (20.0%) | 79 (21.6%) | 0.040 |
| Coronary heart disease, n (%) | 201 (35.6%) | 120 (32.9%) | -0.057 | 141 (38.6%) | 120 (32.9%) | -0.122 |
| Antihypertensive drugs, (n%) | 280 (49.6%) | 183 (50.1%) | 0.012 | 183 (50.1%) | 183 (50.1%) | 0.000 |
| Statin treatment, n (%) | 49 (8.7%) | 22 (6.0%) | -0.111 | 34 (9.3%) | 22 (6.0%) | -0.138 |
| hypoglycemic drugs, n (%) | 82 (14.5%) | 49 (13.4%) | -0.032 | 53 (14.5%) | 49 (13.4%) | -0.032 |

^△^ Standardized Mean Difference

*Notes :* SBP/DBP, systolic/diastolic blood pressure; HDL, high-density lipoprotein; LDL, low-density lipoprotein; TC/HDL, total cholesterol/ high-density lipoprotein; LVMI, left ventricular mass index; CIMT, carotid intima-media thickness; e-GFR, estimated glomerular filtration rate; CF-PWV, carotid-femoral pulse wave velocity; E/Ea, ratio of peek early diastolic transmitral flow velocity(E) and the early diastolic lateral mitral annular velocity(Ea);UACR, urinary albumin-creatinine ratio; LVH, left ventricular hypertrophy; LVDD, left ventricular diastolic dysfunction; PAD, peripheral arterial disease; MAU, microalbuminuria

Values are represented as n (%) or mean ± SD. Optimal DBP (70 to＜80 mmHg), High DBP (80 to＜90 mmHg)


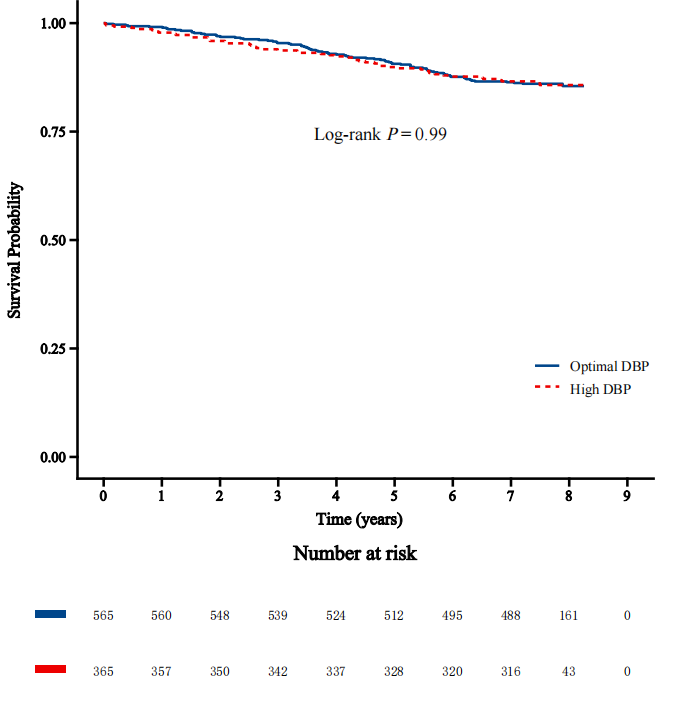

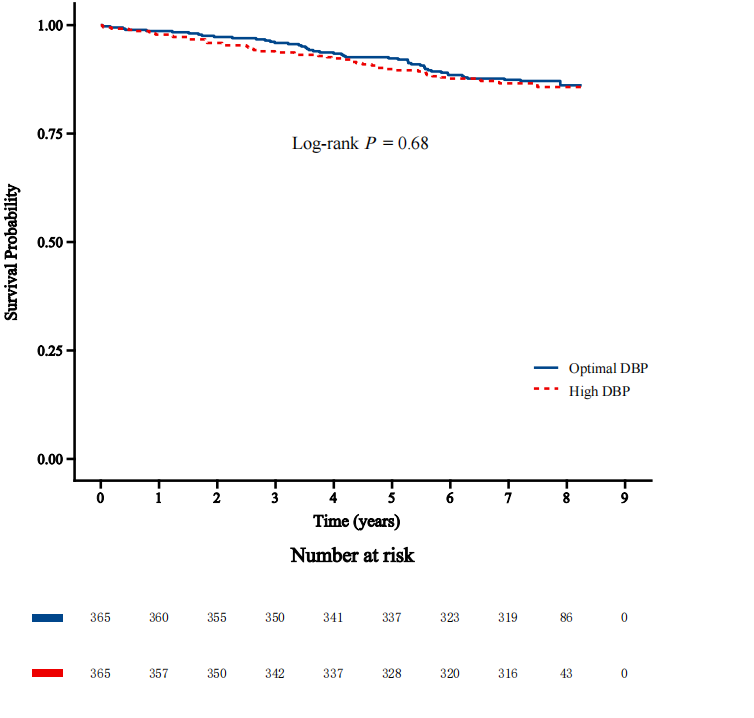


**Fig.S1** Rates of MACEs with DBP before (left) and after (right) matching

*Notes*: MACEs, major adverse cardiovascular events; DBP, diastolic blood pressure

MACEs consisted of all cause death, nonfatal myocardial infarction, and nonfatal stroke. Optimal DBP (70 to < 80 mmHg), High DBP (80 to < 90 mmHg)


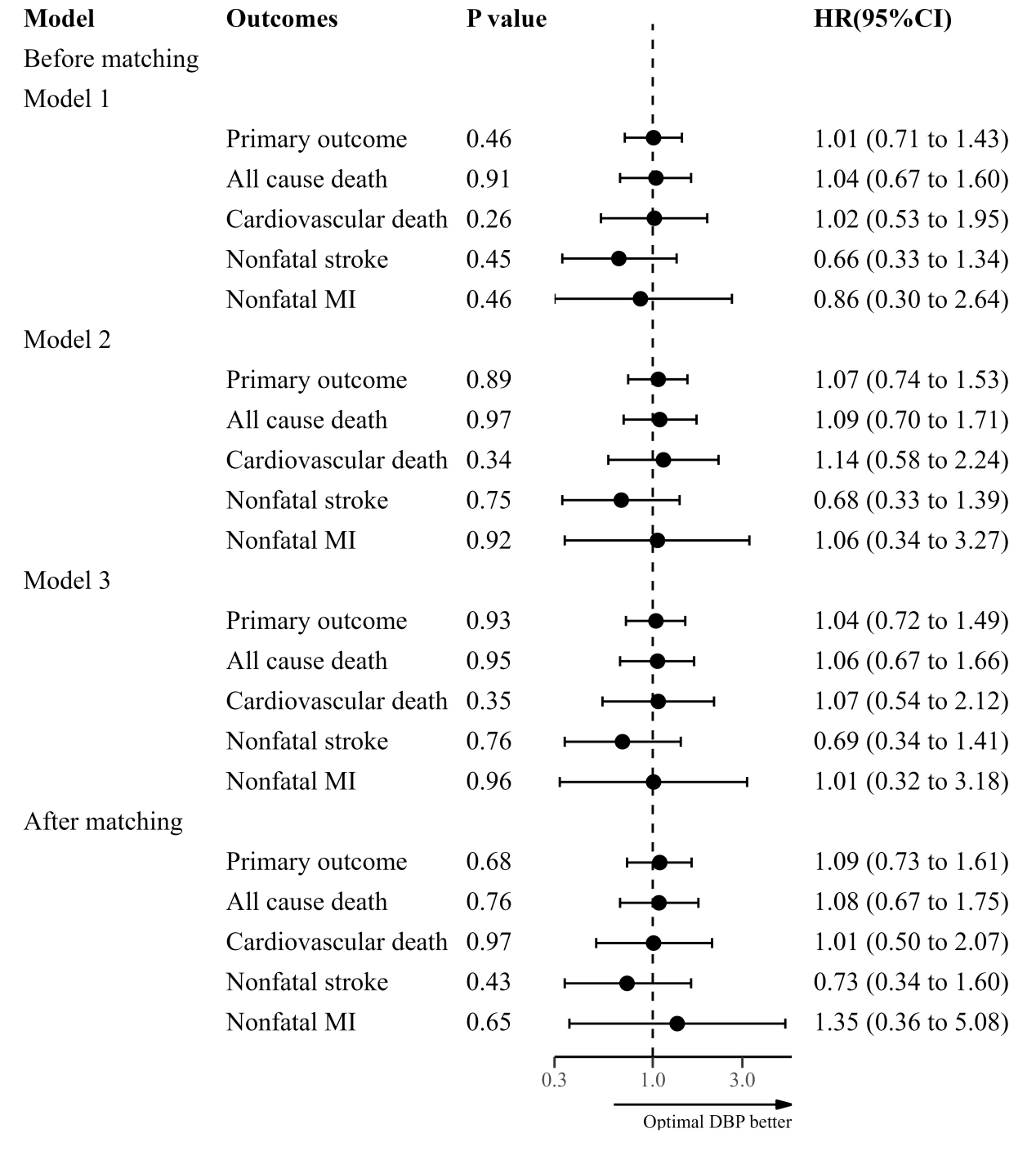


**Fig.S2** Association of MACEs with DBP before and after matching

*Notes:* MACEs, major adverse cardiovascular events; DBP, diastolic blood pressure; MI, myocardial infarction

MACEs consisted of all cause death, nonfatal myocardial infarction, and nonfatal stroke; Model 1 without adjustment; Model 2 with adjustment for age, body mass index, sex, cardiovascular disease history, diabetes, systolic blood pressure, current smoking, triglycerides/high-density lipoprotein ratio. Model 3 including additional adjustments for estimated glomerular filtration rate, antihypertensive treatment, statin treatment, and hypoglycemic treatment in addition to the variables in Model 2. Optimal DBP (70 to < 80 mmHg), High DBP (80 to < 90 mmHg)
